# Chloride of Sodium in Cancer

**Published:** 1829

**Authors:** 


32. *Chloride of Sodium in Cancer.*—Mr. Buchanan, of Hull, (England,) and Mr. Fielding, of the same place, have published cases of Cancer, cured by the internal and external use of the Chloride of Sodium. The Boston Med. and Sur. Journal contains an account of one of these cases, drawn up by the former of those gentlemen. The patient was a female, aged 53, and the disease was seated in the right mamma. After it had existed for several years, and the ulcer was two inches in diameter, with darting pains in the breast, thorax and axilla, it was cured by the application of the tincture of iodine; but the parts remained in a state of considerable induration. In less than a year the whole of the indurated substance was thrown off, leaving a foul, fœtid ulcer, larger than before, with a sanious, bloody, and most offensive discharge, and all her former pains returned, with increased violence. In this state of things recourse was had to the chloride, according to the following formula:—

℞. Liq. Chlor. Sod. ℥vj.

Aq. Distillat. ℥viij. M. fl. Lotio.

" I dipped," says he, "a pledget of lint into this lotion, and applied it to the diseased portion of the breast, with directions to keep the parts constantly moist with it: and also to take two tablespoonsful of the solution three times a day.

"The following day the discharge was changed to the color and consistence of cream, totally divested of its fetid disagreeable smell. The ulcer healed rapidly: the whole of it was soon covered with healthy skin; forming, however, a considerable depression, occasioned by the loss of substance, as if part of the mamma had been dug out. The cure was completed by the latter part of June, 1829, being little more than ten or twelve days from the time of the first application of the solution of the chloride, and with only six bottles of the above, which were used indiscriminately as mixture and lotion. The patient was employed in the harvest following, and as she expressed it, "wrought in better health and spirits than she had done for these last twenty years."

